# Mortality in ASIA Impairment Scale grade A to D Patients With Odontoid Fracture and Magnetic Resonance Imaging Evidence of Spinal Cord Injury

**DOI:** 10.1089/neur.2023.0005

**Published:** 2023-06-01

**Authors:** Bizhan Aarabi, Christopher J. Neal, David S. Hersh, James S. Harrop, Michael G. Fehlings, Elizabeth G. Toups, James D. Guest, Beatrice Ugiliweneza, Noori Akhtar-Danesh, Shekar N. Kurpad, Robert G. Grossman

**Affiliations:** ^1^ Department of Neurosurgery, University of Maryland School of Medicine, Baltimore, Maryland, USA.; ^2^ Department of Neurosurgery, Walter Reed National Military Medical Center, Bethesda, Maryland, USA.; ^3^ Division of Neurosurgery, Connecticut Children's, Hartford, Connecticut, USA.; ^4^ Department of Neurosurgery, Jefferson Medical College, Philadelphia, Pennsylvania, USA.; ^5^ Division of Neurosurgery and Spine Program, University of Toronto, Toronto, Ontario, Canada.; ^6^ Department of Neurosurgery, University of Texas, Houston at Methodist Hospital, Houston, Texas, USA.; ^7^ Department of Neurological Surgery, the Miami Project to Cure Paralysis, Miami, Florida, USA.; ^8^ Department of Neurosurgery, Kentucky Spinal Cord Injury Research Center, Louisville, Kentucky, USA.; ^9^ School of Nursing and Department of Health Research Methods, Evidence, and Impact, McMaster University, Hamilton, Ontario, Canada.; ^10^ Department of Neurosurgery, Medical College of Wisconsin, Milwaukee, Wisconsin, USA.

**Keywords:** magnetic resonance imaging, odontoid fractures, outcome, trauma

## Abstract

Odontoid fractures are common, often presenting in the elderly after a fall and infrequently associated with traumatic spinal cord injury (tSCI). The goal of this study was to analyze predictors of mortality and neurological outcome when odontoid fractures were associated with signal change on magnetic resonance imaging (MRI) at admission. Over an 18-year period (2001–2019), 33 patients with odontoid fractures and documented tSCI on MRI were identified. Mean age was 65.3 years (standard deviation [SD] = 17.2), and 21 patients were male. The mechanism of injury was falls in 25 patients, motor vehicle accidents in 5, and other causes in 3. Mean Injury Severity Score (ISS) was 40.5 (SD = 30.2), Glasgow Coma Scale (GCS) score was 13 (SD = 3.4), and American Spinal Injury Association (ASIA) motor score (AMS) was 51.6 (SD = 42.7). ASIA Impairment Scale (AIS) grade was A, B, C, and D in 9, 2, 3, and 19 patients, respectively. Mean intramedullary lesion length was 32.3 mm (SD = 18.6). The odontoid peg was displaced ventral or dorsal in 15 patients. Twenty patients had surgical intervention: anterior odontoid screw fixation in 7 and posterior spinal fusion in 13. Eleven (33.3%) patients died in this series: withdrawal of medical care in 5; anoxic brain injury in 4; and failure of critical care management in 2. Univariate logistic regression indicated that GCS score (*p* < 0.014), AMS (*p* < 0.002), AIS grade (*p* < 0.002), and ISS (*p* < 0.009) were risk factors for mortality. Multi-variate regression analysis indicated that only AMS (*p* < 0.002) had a significant relationship with mortality when odontoid fracture was associated with tSCI (odds ratio, 0.963; 95% confidence interval, 0.941–0.986).

## Introduction

Odontoid fractures are one of the most common types of cervical spine fractures observed.^1^ Classically, odontoid fractures present in the elderly after a fall, often associated with striking the head and resulting in a hyperextension of the upper cervical spine.^2^ Though only ∼20% of cervical traumatic (tSCI) spinal cord injuries (SCIs) are centered around odontoid fractures, mortality in this group of patients is unusually high (∼50%) compared to the 15% mortality associated with cervical tSCI in general.^3,4^ When neurological deficits do occur, they are likely secondary to displacement of the odontoid process, with resultant spinal cord compression.^5–9^

The goal of this investigation was 3-fold, including to, first, identify the mortality in odontoid fractures associated with SCI and, second, better understand the rate of American Spinal Injury Association (ASIA) Impairment Scale (AIS) grade conversion post-tSCI at the level of the odontoid peg during at least 6 months of follow-up. Last, we investigated whether the mortality rate in odontoid fractures was associated with neurological deficit as confirmed by magnetic resonance imaging (MRI).

## Methods

### Study design

This work was a retrospective study of a prospectively collected database.

### Primary objective

Mortality in patients with odontoid fracture and neurological deficit was the primary objective.

### The cohort

From January 2001 to January 2019, a total of 2176 patients with the primary diagnosis of tSCI were admitted to this level 1 trauma center. From this cohort, 405 (18.7%) patients sustained upper cervical spine fracture dislocations whose SCI was confirmed by MRI (occipital condyle, C1 and C2): C1, 13; body of C2, 13; occipito-atlanto-axial vertical distraction, 58; hangman fracture, 60; combination fractures, 76; and Anderson D'Alonzo types 1, 2, and 3 fractures in 185. Atlas, body, hangman, and combination fractures and vertical distraction injuries were excluded from this study. In addition, patients with no visible signal on MRI, penetrating SCI, traumatic brain injury, not testable International Standards for Neurological Classification of Spinal Cord Injury (ISNCSCI), or tumors and those with no hospital charts or inadequate follow-up information were excluded. The study started after DoD Human Research Protection Office and institutional review board registration and review (Table 1).

**Table 1.** Basic Clinical Characteristics of a Cohort of 33 Patients With Odontoid Fracture and tSCI

Case no.	Age	Mech	Pres	Gen	GCSs	ISS	AMS	AIS grade	Head CT	DISPL	IMLL	FAMS	FAISg	Cause of death
1	78	GLF	ATCCS	0	15	10	100	D	NL	0	X	98	D	NA
2	80	GLF	TETRA	1	11	75	0	A	NL	1	12.2	NA	NA	Acute care
3	57	GLF	ATCCS	1	15	X	55	C	NL	1	25.7	96	D	NA
4	57	Sports	TETRA	1	15	75	0	A	NL	0	32	0	A	Withdrawal of care
5	62	GLF	TETRA/coma	1	3	75	0	A	NL	1	23.1	NA	NA	ABI
6	24	MVC	ATCCS	0	14	17	15	C	NL	0	16.5	95	D	NA
7	86	GLF	ATCCS	0	15	X	71	D	NL	0	57.2	100	D	NA
8	41	MVC	ATCCS	1	15	18	58	D	NL	0	52.3	95	D	NA
9	18	MVC	TETRA	1	10	75	0	B	NL	1	11.7	100	D	NA
10	55	GLF	TETRA	1	11	75	0	A	NL	0	22.4	0	A	NA
11	53	SPORTS	Tetra/coma	1	6	75	0	A	NL	0	25.1	NA	NA	Acute care
12	44	GLF	ATCCS	1	15	X	98	D	NL	0	35.7	100	E	NA
13	84	GLF	ATCCS	1	15	4	100	D	NL	0	64.3	100	D	NA
14	86	GLF	ATCCS	0	15	X	92	D	NL	1	40.8	90	D	NA
15	63	GLF	ATCCS	1	15	9	92	D	NL	1	14.1	95	D	NA
16	71	GLF	MP	1	15	4	95	D	NL	1	31.9	99	D	NA
17	86	GLF	TETRA	1	15	X	74	D	NL	0	77.8	UNK	UNK	Withdrawal of care
18	73	GLF	NS	0	15	26	94	D	NL	0	18.2	100	E	NA
19	81	GLF	TETRA	1	15	X	80	D	NL	1	19.8	100	D	NA
20	81	MVC	TETRA/coma	1	9	16	0	B	NL	0	17.8	5	B	ABI
21	52	GLF	ATCCS	0	15	24	86	D	NL	0	13.8	100	E	NA
22	44	MVC	ATCCS	0	15	20	74	D	NL	0	48.1	100	D	NA
23	74	GLF	TETRA	0	11	75	0	A	NL	0	54.5	19	A	NA
24	62	GLF	TETRA	0	15	75	0	A	NL	0	16.8	NA	NA	Withdrawal of care
25	72	GLF	TETRA	1	15	24	94	D	NL	1	44	94	D	Withdrawal of care at 3 months
26	74	GLF	ATCCS	0	15	13	96	D	NL	1	31.3	100	D	NA
27	65	GLF	MP	1	15	17	97	D	NL	0	15	97	D	NA
28	67	GLF	HP	1	15	X	82	D	NL	0	51.2	88	D	NA
29	59	GLF	TETRA	0	11	75	0	A	NL	1	X	0	A	ABI
30	71	GLF	ATCCS	1	15	X	91	D	NL	1	60	75	D	NA
31	81	GLF	TETRA	0	11	75	3	C	NL	1	X	NA	NA	Withdrawal of care
32	85	GLF	TETRA/coma	1	3	X	0	A	NL	1	29.6	0	A	ABI
33	68	Other	HP	1	15	20	57	D	NL	1	6.7	83	D	NA

ABI, anoxic brain injury; AIS, ASIA Impairment Scale; AMS, ASIA motor score; ATCCS, acute traumatic central cord syndrome; CT, computed tomography; DISPL, displacement; FAISg, follow-up AIS grade; FAMS, follow-up AMS; GLF, ground-level fall; HP, hemiparesis; IMLL, intramedullary lesion length; ISS, Injury Severity Score; Mech, mechanism; NA, not applicable; MVC, motor vehicle crash; MP, monoparesis; NL, normal; NS, not specified; Pres, presentation; TETRA, tetraparesis/tetraplegia; tSCI, traumatic spinal cord injury; UNK, unknown .

### Initial resuscitation

Patients were transferred to the trauma center by the members of the emergency medical technicians (EMTs) and, when deemed medically stable, examined by the neurosurgical team, including the senior resident, nurse practitioners, and attending neurosurgeon. A complete neurological evaluation was performed according to the ISNCSCI, including ASIA motor score (AMS), AIS grade, and neurological level of injury.

### Imaging studies

A multi-planar cervical spine computed tomography (CT) scan was performed within 2–4 h of trauma, and multi-sequence, multi-planar T2-weighted and short T1 inversion recovery (STIR) sequences were acquired within 5–7 h post-trauma.^10^ We defined the Anderson D'Alonzo class of the odontoid fracture and the Benzel classification of the C2 body fracture.^11,12^ In addition, the degree of displacement of the odontoid process in the spinal canal was determined. Intramedullary lesion length (IMLL) was measured on sagittal T2 and/or STIR MRI.^13^

### Steroid protocol

Between 2001 and 2009, study patients were administered methylprednisolone post-SCI—30 mg/kg within the first hour and 5.4 mg/kg/h for the next 23 h. Starting in 2010, the use of steroids for SCI was discontinued.^14^ Patients' mean arterial blood pressure was maintained between 85 and 90 mm Hg for 7 days post-trauma, when medically feasible.^15^

### Traction reduction and surgical realignment and fusion

When the odontoid peg was displaced, traction reduction was performed and surgical stabilization was carried out, when indicated. Anterior odontoid screw fixation or posterior spinal fusion was performed, when indicated.

### Intensive care unit care and long-term follow-up

In the intensive care unit (ICU), enoxaparin (Lovenox^®^; Sanofi, Bridgewater, NJ) was administered 30 mg twice-daily starting within 24–48 h of trauma for deep vein thrombosis (DVT) prophylaxis, with screening for DVT by duplex ultrasound, when clinically indicated. Early tracheostomy for ventilator support and percutaneous gastroenterostomy for nutrition were also routinely carried out, when indicated, as determined by the ICU and trauma team. During the entire in-hospital stay, ISNCSCI examinations, including digital rectal examination, were performed each day to track changes in AMS and AIS grade. After discharge, patients returned to the clinic at 6 weeks, 3 months, 6 months, and 12 months (or longer) for follow-up ISNCSCI examinations, which were performed by the staff of the Department of Neurosurgery as well as certified neurologists and rehabilitation specialists in other facilities.

### Statistical analysis

Descriptive statistics of mean and standard deviation (SD) are reported for continuous variables whereas frequencies and proportions are reported for categorical variables. Associations between any categorical variables and mortality were examined using a chi-square test. We used the two-sample independent *t*-test to compare any continuous variables between patients who died and those who survived. Finally, multiple logistic regression was used to assess the relationship between mortality and independent variables and results. The Stata SE/12.1 statistical program (StataCorp, College Station, TX) was used for all analyses.

## Results

### Demographics

Twelve of 33 patients in this study were females (36.4%). Mortality in women was 25% and in men 38.1%. This difference was not significant (*p* = 0.443). Mean age in this cohort was 65.3 years (SD = 17.3; range, 18–86). Mean age in the patients who survived was 62.5 years (SD = 18.9); in patients who died, it was 70.7 years (SD = 12.4). The difference was not significant (*p* = 0.20; Table 2).

**Table 2.** Baseline Characteristics of Patients Who Survived vs. Those Who Died After Odontoid Fracture and tSCI

Characteristic	Patients who survived (*n* = 22)	Patients who died (*n* = 11)	*p* value
Sex			0.443
Male	13 (61.9)	8 (38.1)	
Female	9 (75)	3 (25)	
Mean age (years)	62.5 (18.9)	70.7 (12.3)	0.204
Mechanism of injury (%)			0.471
Fall	17 (68)	8 (32)	
MVC	4 (80)	1 (20)	
Other	1 (33.3)	5 (66.7)	
Mean admission GCS (SD)	14.36 (1.52)	10.36 (4.65)	0.0008
Mean admission ISS (SD)	27.13 (25.58)	62.77 (8.11)	0.0028
Mean comorbidities (SD)	2.88 (2.42)	3.88 (2.02)	0.297
AIS grade A and B C and D	3 (27.27) 19 (86.36)	8 (72.73) 3 (13.64)	<0.001
Displaced odontoid process Yes No	9 (60) 13 (72.2)	6 (40) 5 (27.8)	0.550
ASIA motor score, mean (SD)	69.68 (34.7)	15.54 (34.15)	<0.0002
IMLL, mean (SD)	32.91 (18.42)	30.93 (19.96)	0.794

AIS, ASIA Impairment Scale; ASIA, American Spinal Injury Association; GCS, Glasgow Coma Scale; IMLL, intramedullary lesion length; ISS, Injury Severity Scale; MVC, motor vehicle crash; SD, standard deviation; tSCI, traumatic spinal cord injury.

### Mechanism of injury

Twenty-five patients with odontoid fractures suffered from falls, of which 8 died (32%). On the other hand, 4 of 5 patients who had motor vehicle collisions and sustained odontoid fractures survived (80%). Mechanism of accident in odontoid fractures was not efficacious in mortality (*p* = 0.52; Table 2).

### Comorbidities, Injury Severity Score

**FIG. 1.**
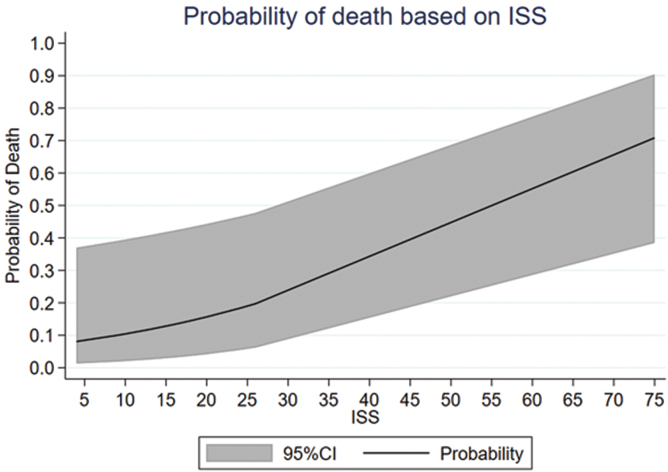
Graph indicating the probability of death during acute care and at least 6 months of follow-up in patients with tSCI attributable to Anderson D'Alonzo type II and III odontoid fractures as the Injury Severity Score (ISS) increased. The effect size of ISS on death rate was significant (*p* = 0.002). 95% CI, 95% confidence interval; tSCI, traumatic spinal cord injury .

Mean comorbidity score was 3.2 (SD = 2.3). Mean Injury Severity Score (ISS) was 40.5, (SD = 30.2). Although comorbidity score did not differ between patients who died and those who survived (*p* = 0.29), ISS was significantly higher in patients who died after admission (*p* = 0.002; Table 2; Fig. 1).

### Glasgow Coma Scale score

**FIG. 2.**
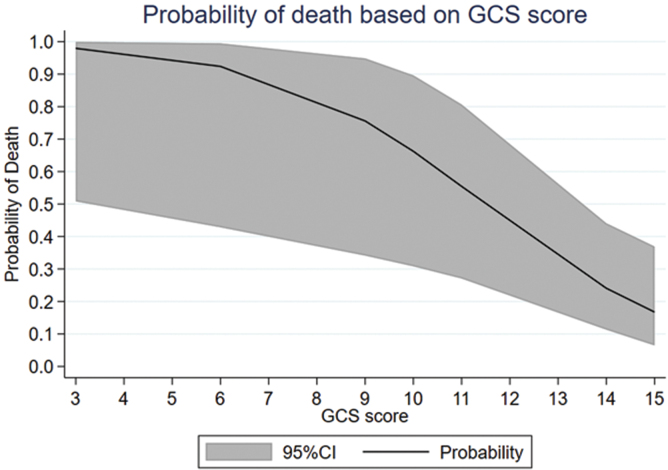
Graph indicating the probability of death during acute care and at lease 6-month of follow-up in patients with tSCI attributable to Anderson D'Alonzo type II and III odontoid fractures as admission Glasgow Coma Scale score is decreased. The effect size of GCS score on death rate was highly significant (*p* = 0.0008). 95% CI, 95% confidence interval; tSCI, traumatic spinal cord injury.

Mean Glasgow Coma Scale (GCS) score in 33 patients with odontoid fractures was 13.0 (SD = 3.4). GCS score was 3–8 in 3, 9–12 in 7, and 13–15 in 23 patients. There was a strong correlation between GCS score at the time of admission and mortality (*p* = 0.0008; Table 2; Fig. 2).

### ASIA motor score

**FIG. 3.**
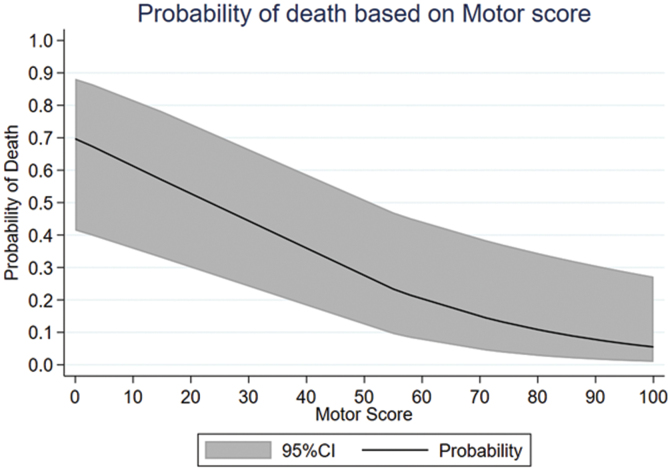
Graph indicating the probability of death during acute care and at least 6 months of follow-up in patients with tSCI attributable to Anderson D'Alonzo type II and III odontoid fractures as there was a drop in ASIA motor score. The effect size of AMS on death rate was significant (*p* < 0.0002). In multiple logistic regression analysis, the effect size of AMS and mortality was more pronounced (odds ratio, 0.963; 95% CI, 0.941–0.986; *p* = 0.001). 95% CI, 95% confidence interval; AMS, ASIA motor score; ASIA, American Spinal Injury Association tSCI, traumatic spinal cord injury.

AMS was available in 33 patients (mean, 51.6; SD = 42.7). AMS in 22 patients who survived was 69.6 (SD = 34.7), and in 11 patients who died it was 15.5 (SD = 34.1; *p* < 0.0002; Fig. 3).

### ASIA Impairment Scale grade

Only 3 of 11 patients with AIS grade A/B survived whereas 19 of 22 patients with AIS grade C and D had long-term survival (*p* = 0.001).

### Computed tomography and magnetic resonance imaging morphology and morphometry

Morphological assessment of the cervical spine was determined by multi-planar CT, and multi-planar, multi-sequence MRI determined the Anderson D'Alonzo fracture type, displacement of the odontoid process, and length of the IMLL. Accordingly, 23 of 33 patients had type II and 10 patients had type III odontoid fractures (where fracture line transgresses into the C1/C2 joints). The odontoid peg was displaced anteriorly or posteriorly in 15 and was not displaced in 18 patients. As described in the reports by Patel and colleagues^4^ and Harrop and colleagues,^16^ displacement of the odontoid process did not increase chances of mortality. On T2-weighted images or T1 inversion recovery, the IMLL ranged from 6.7 to 77.8 mm (mean, 32.3; SD = 18.6). IMLL in patients who survived was 32.9 mm (SD = 18.4), and in patients who did not survive their injury, it was 30.9 mm (SD = 19.9). The difference was not statistically significant (*p* < 0.794).

### Surgical intervention

Seven of 33 patients with odontoid fractures had anterior odontoid screw fixation with 2 deaths. Mortality for the 13 patients with posterior spinal fusion was 38.5%. Non-operative management was considered in 13 patients, of which 4 died.

### Outcome

**FIG. 4.**
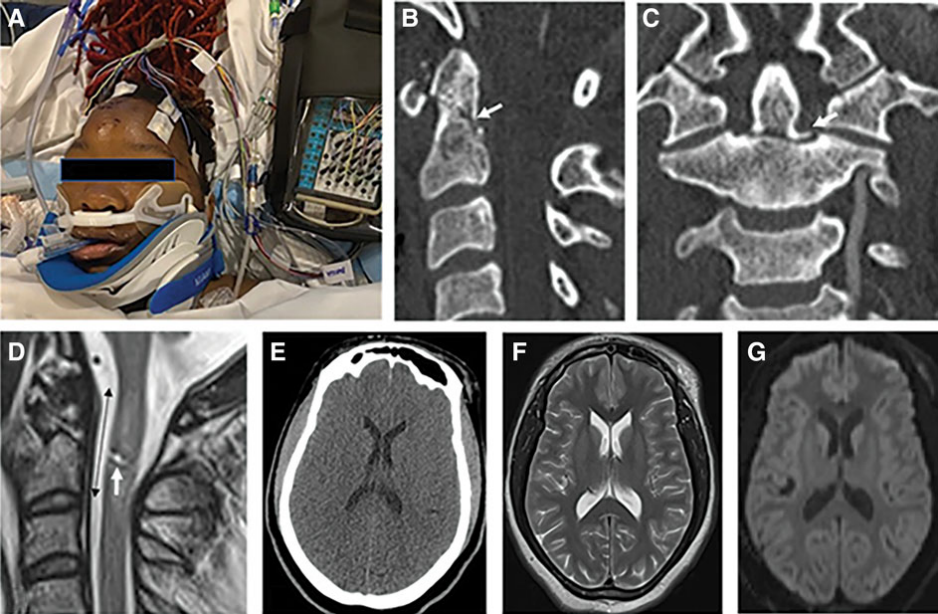
This 33-year-old woman was an unbelted rear-seat passenger during a motor vehicle accident. At the scene of the accident, the emergency medical technicians found her in complete cardiorespiratory arrest. After resuscitation, she was airlifted to the trauma center. In the trauma center, she had one more episode of cardiac arrest followed by continuous facial myoclonus (**A**). Her Glasgow Coma Scale score was 3; pupils were small and minimally reactive. There were no corneal reflexes, and cold calorics did not induce any horizontal movement of the eyeballs. CT scan of the cervical spine showed type II odontoid fracture with minimal displacement (**B,C**). T2-weighted MRI images indicated near transection of the spinal cord immediately behind the injury epicenter and spinal cord swelling. Intramedullary lesion length was 28.8 mm (**D**). Brain CT and MRI were negative for TBI and ischemic stroke (**E,F,G**), except for a focal point of infarction in the cerebellum attributable to blunt cerebrovascular injury to the right vertebral artery (**C**). Diffusion tensor images did not reveal any evidence of restricted diffusion in the basal ganglia or cortex (**G**). CT, computed tomography; MRI, magnetic resonance imaging; TBI, traumatic brain injury.

As indicated in Tables 1 and 2, from 11 patients who did not survive after odontoid fracture and tSCI, 4 suffered from anoxic brain injury (Fig. 4), 5 had withdrawal of support, and 2 died from medical complications. Eight of 11 patients with motor-complete tSCI died (74%; 1 AIS grade B and 7 AIS grade A). From the 4 patients with anoxic brain injury, 2 died, 2 and 3 months after discharge. From the 5 patients with withdrawal of support, 1 patient died 18 months and 2 each 3 months after discharge.

Long-term follow-up of 22 patients who survived their odontoid fracture indicated AIS grade conversion in 8 patients (36.3%; Table 3).

**Table 3.** AIS Grade Conversion in 22 Patients Who Had tSCI After Odontoid Fracture

	No.	A	B	C	D	E	Total
A	2	1	1	0	0	0	2
B	1	0	0	0	1	0	1
C	2	0	0	0	2	0	2
D	17	0	0	0	13	4	17

AIS, American Spinal Injury Association Impairment Scale; tSCI, traumatic spinal cord injury.

**Table 4.** Univariate Analysis of Determinants of Mortality

Outcome	Odds ratio	*p* value	95% confidence interval
AIS	0.0592105	0.002	0.0097784–0.3585349
ISS	1.047844	0.009	1.011603–1.085383
GCS score	0.6341018	0.014	0.441633–0.910457
AMS	0.939389	0.002	0.7064954–7.471983

AIS, American Spinal Injury Association Impairment Scale; ISS, Injury Severity Score; GCS, Glasgow Coma Scale; AMS, ASIA motor score.

**Table 5.** Determinants of Mortality in Multiple Logistic Regression Model

Outcome	Odds ratio	*p* value	95% confidence interval
ASIA motor score	0.9639489	0.002	0.9414697–0.986965
_conc	2.29759	0.169	0.7064954–7.471983

ASIA, American Spinal Injury Association.

## Discussion

This investigation presented evidence that: 1) When odontoid fractures were associated with tSCI, they were unusually deadly. Long-term survival was particularly compromised in AIS grade A and B quadriplegics with a low AMS. 2) IMLL did not have a significant relationship with mortality. 3) One or two levels upward AIS grade conversion was observed in 35% of patients who survived their injury for at least 6 months of follow-up.

**FIG. 5.**
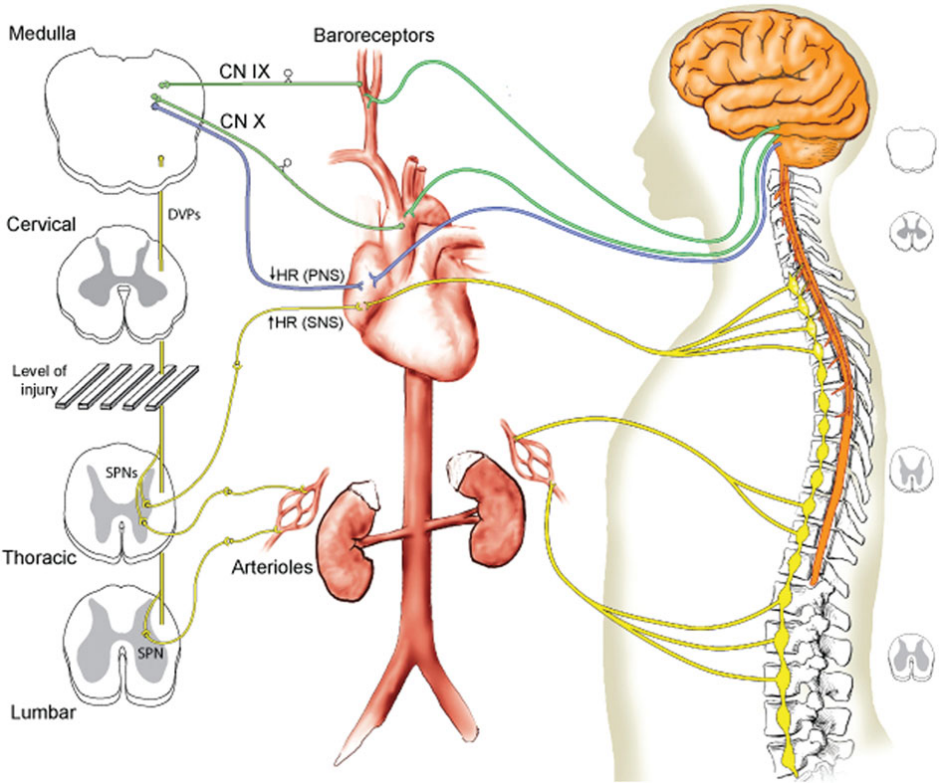
Schematic representation of the central and autonomic innervation of the cardiovascular system. On the left (front view of spinal cord), the spinal sympathetic pre-ganglionic neurons (SPNs) are shown as disconnected from the medullary cardiovascular centers by a cervical SCI, whereas the parasympathetic nervous system (PNS) is intact. Parasympathetic cardiac innervation (in blue) and sympathetic cardiac innervation (in yellow) can reduce and increase heart rate (HR), respectively. Parasympathetic afferents from baroreceptors of the aortic arch and carotid artery travel to the medulla oblongata through cranial nerves (CN) IX (glossopharyngeal nerve; in green) and X (vagus nerve; in green). Efferent fibers are denoted by yellow or blue lines, afferent fibers by green lines. The right-sided figure schematically depicts the neuroanatomy of the cardiovascular innervation, including the central nervous system (orange), extramudullary SNS (yellow), and extramudullary PNS (blue). From Furlan and Fehlings,^20^ with permission. DVPs, descending vasomotor pathways; SCI, spinal cord injury; SNS, sympathetic nervous system.

The mechanism responsible for such a higher mortality rate (33.3%) than the mortality rate reported in subaxial tSCI patients is thought to involve autonomic dysregulation and cardiopulmonary abnormalities that accompany SCI at a high segmental level (Fig. 5). From the 17 patients with neurological impairment evaluated by Harrop and colleagues,^16^ 11 required cardiopulmonary resuscitation at the scene of the accident. Cervical tSCI at the level of the craniocervical junction resulted in quadriplegia and loss of diaphragmatic function.

Disruption of involuntary ascending Hering-Breuer signals to the medullary respiratory centers and interruption of descending lateral medullary reticulospinal tract regulatory motor pathways into the diaphragmatic and thoracic motor cell columns resulted in sudden respiratory arrest and loss of ventilation.^20^

In addition, not only did loss of thoracolumbar sympathetic signals lead to neurogenic shock, but also overactivity of parasympathetic signals by the vagus nerve brought about bradyarrhythmias followed by cardiac arrest at the scene of accident, during transfer, or in the trauma resuscitation unit.^2^ Therefore, the patients were admitted in total cardiorespiratory arrest, coma, and myoclonus attributable to cerebral hypoxia. Four of 11 patients in our series had evidence of anoxic brain injury. In the Patel and colleagues series, 9 of 10 patients who did not survive their neurological injury died from cardiac or cardiorespiratory events.^4^

In subaxial tSCI studies, longer IMLL had a negative prognosticating effect on AIS grade conversion.^13^ In the present investigation, IMLL did not differ between patients who died and those who survived their tSCI subsequent to odontoid fractures. This finding should be validated in future studies.

In the present investigation, acute care could not save 2 patients who died 6 and 11 days after admission. Another 5 patients died after having their life support withdrawn either by themselves or their legally authorized representative during acute care (2 patients) or 18 months after discharge in 1 and 3 months after discharge each in 1 patient. National Crash Severity Study data reported by Huelke and colleagues^18^ indicated that 20% of all in-car deaths include fatal cervical spine injuries, a finding previously reported by Bucholz and colleagues.^21^ Nearly 80% of these fatal cervical spine injuries are centered in the upper cervical spine, particularly C2 fractures. As suggested in the pathophysiological report by Furlan and colleagues (Fig. 5),^20^ one major reason for sudden death is neurogenic shock, bradyarrhythmias, and anoxic brain injury. Four of 11 patients in our series presented obtunded or stuporous, constant facial myoclonus, and in a few status epilepticus confirmed by electroencephalography and thus indicative of anoxic brain injury (Fig. 4). These patients had normal head CT scans. EMTs quickly intubated these patients and subsequently transferred them to the trauma center within the hour subsequent to tSCI.^22^ When confronted with anoxic brain injury, it is an option to apply hypothermia^23,24^ and extracorporeal membrane oxygenation in order to combat global hypoxia.^25^ Ventilator support and its complications are some of the reasons for drastic decisions to withdraw support. Thus, early evaluation and implantation of diaphragmatic pacing systems could add to the prospects of early discharge and improvements in long-term outcome.^26,27^ Presence of coma at admission and difficulty with long-term ventilator support during acute care hospitalization were previously reported in the Patel and Harrop studies.^4,16^

## Conclusion

tSCIs subsequent to odontoid fractures can be divided into two distinct groups of patients: One group has primarily incomplete tSCI in the form of acute traumatic central cord syndrome (ATCCS) with almost no mortality; the second group suffers from anoxic brain injury or complete quadriplegia. Mortality in these two groups of patients is unacceptably high. Recognizing this subset of patients who present with tSCI and an odontoid fracture can further aid in triage, the decision to intervene surgically, and appropriate counseling of families. Evidence recommends earlier anatomical realignment and internal fixation (anterior odontoid screw fixation or C1/C2 posterior spinal fusion) in incomplete cervical SCI patients with prospects for good functional outcome and conservative management in patients with anoxic brain injury whose prospects for survival and functional recovery are poor.

### Limitations

The evidence presented in this investigation is retrospective analysis of prospectively collected clinical data from a single level 1 trauma center.

## Abbreviations Used

AIS: ASIA Impairment Scale
AMS: ASIA motor score
ASIA: American Spinal Injury Association
ATCCS: acute traumatic central cord syndrome
CT: computed tomography
DVT: deep vein thrombosis
EMTs: emergency medical technicians
GCS: Glasgow Coma Scale
ICU: intensive care unit
IMLL: intramedullary lesion length
ISNCSCI: International Standards for Neurological Classification of Spinal Cord Injury
ISS: Injury Severity Scale
MRI: magnetic resonance imaging
SCI: spinal cord injury
SD: standard deviation
STIR: short T1 inversion recovery
tSCI: traumatic spinal cord injury
